# The Preliminary Efficacy and Feasibility of SMILE Serious Game for Adolescents and Young Adults: Protocol for Cluster Randomized Multisite Adaptive Trial Design

**DOI:** 10.2196/96292

**Published:** 2026-09-03

**Authors:** Ewelina Smoktunowicz, Jan Maciejewski, Magdalena Lesnierowska, Magdalena Pietrzak, Franziska Großwendt, Gwendolyn Mayer, Jobst-Hendrik Schultz, Nelli Tschobur, Mel McKendrick, Helen Stocks, Francesca Floris, Dina Guglielmi, Zouhair Haddi, Carlo Mazzola, Izidor Mlakar, Urška Smrke, Laura Fernández García, Esther Parra Vidales, Elisa Sáez Muñoz, Sebastian Bruchhaus, Matthias Hemmje, Dominic Heutelbeck, Dah Diarra, Habib Nasser, Alex Butean, Marco Polak, Panagiota Mourettou, Angelos Parmatzias, Ula Kolinska, Matthias Schwannauer

**Affiliations:** 1 StresLab Research Center, Institute of Psychology, SWPS University, Chodakowska 19/31, Warsaw, 03-815, Poland, 48 225179921; 2 Institute of Psychology, SWPS University, Warsaw, Poland; 3 Department of General Internal and Psychosomatic Medicine, University Hospital Heidelberg, Heidelberg, Baden-Wurttemberg, Germany; 4 School of Social Sciences, Heriot-Watt University, Edinburgh, Scotland, United Kingdom; 5 Department of Education Studies, University of Bologna, Bologna, Italy; 6 Digital Health Department, NVISION Systems and Technologies, Barcelona, Spain; 7 Faculty of Electrical Engineering and Computer Science, University of Maribor, Maribor, Slovenia; 8 Fundación INTRAS, Valladolid, Castille and León, Spain; 9 Research Institute for Telecommunication Cooperation, Dortmund, North Rhine-Westphalia, Germany; 10 RDIUP, Les Mureaux, France; 11 WIZ Development and Services SRL, Sibiu, Romania; 12 Lucian Blaga University of Sibiu, Sibiu, Romania; 13 DigitalTwin Technology GmbH, Cologne, Germany; 14 Citizens in Power, Nicosia, Cyprus; 15 School of Health in Social Science, University of Edinburgh, Edinburgh, Scotland, United Kingdom

**Keywords:** serious games, adolescent mental health, adaptive trial, cognitive behavioral therapy, mHealth

## Abstract

**Background:**

Mental health problems frequently emerge during adolescence and young adulthood, yet many young people do not seek or receive timely support. Digital mental health interventions, including serious games and gamified tools, may improve accessibility and engagement, but evidence for their feasibility and preliminary efficacy across diverse European settings remains limited.

**Objective:**

This protocol describes the evaluation and trial component of the SMILE (Supporting Mental Health in Young People: Integrated Methodology for Clinical Decisions and Evidence-Based Interventions) project, which aims to evaluate the acceptability, feasibility, reach, and preliminary efficacy of a cognitive behavioral therapy (CBT)–informed gamified mobile intervention for adolescents and young adults, and to explore mechanisms of change and the impact of its implementation contexts.

**Methods:**

SMILE uses a cluster-randomized, multisite, multiarm adaptive proof-of-concept design across 7 European countries, complemented by semistructured stakeholder interviews. Adolescents and young adults aged 10‐24 years are recruited mainly through schools, universities, and community channels; the target sample is 1438 participants. Clusters are assigned to 1 of 3 sequences comprising a neutral baseline period followed by 2, 4, or 5 game modules, and are randomized to a feedback or no-feedback version of the intervention. The intervention consists of the SMILE Game App (Nurogames and DigitalTwin Technology) and Companion App (RDIUP), which deliver CBT-based gamified content, self-monitoring, experience sampling, and, in one of the study conditions, automated personalized feedback. Primary outcomes are anxiety and depression. Secondary outcomes include well-being, resilience, emotion regulation, self-efficacy, and social anxiety. Feasibility, acceptability, engagement, app usage, in-game metrics, and diary-based digital markers are also assessed. Quantitative data will be analyzed using linear mixed-effects models with adaptive interim analyses. Qualitative stakeholder interviews with young people, their legal guardians, school professionals, and clinicians will be analyzed using inductive thematic analysis within a mixed-methods realist evaluation framework.

**Results:**

The proof-of-concept trial was prospectively registered on April 29, 2025 (ISRCTN32523126). Study rollout is taking place from September 2025 to June 2026. As of March 2026, recruitment is ongoing, 2 study sequences have been completed, and the third sequence began at the end of March 2026. Primary results are expected in September 2026.

**Conclusions:**

SMILE is designed to generate evidence on the feasibility, acceptability, and preliminary efficacy of a scalable gamified mental health intervention for young people across diverse European contexts. The findings will inform intervention refinement, evaluation of the added value of personalized feedback, and the design of a future definitive randomized controlled trial and broader implementation in educational and clinical settings.

## Introduction

### Background

The World Health Organization (WHO) defines mental health as “a state of mental well-being that enables people to cope with the stresses of life, realize their abilities, learn and work well, and contribute to their community. It has intrinsic and instrumental value and is a basic human right” [1]. This definition highlights that mental health is more than just the absence of a disorder; it is a major global health concern, with an estimated 20% of disability-adjusted life years (DALYs) worldwide in 2023 [2]. Adolescence is widely recognized as a sensitive period for the development of mental disorders [3-5], whose incidence has been increasing in recent decades [5-8]. The WHO specifically emphasizes the poor mental health among young people, who often struggle to access mental health services and avoid seeking professional help due to fear of stigmatization [9]. Consequently, these disorders often remain undetected and untreated until later in life [2,10]. Depression is highly prevalent among young people worldwide [2,3,5,7,8,11], and suicide is one of the leading causes of death among European adolescents [10-13].

Prevention recommendations for improving adolescent mental health include connecting with other people, self-help strategies after self-harm, accessibility of care, and a supportive school environment [6-8,14,15]. In the wake of the COVID-19 pandemic, which imposed contact restrictions, online interventions for preventive measures have been developed [16]. Initial research indicates that these interventions have the potential to significantly prevent increasing depression scores; however, more evidence is needed regarding their efficacy in addressing anxiety and stress [14,17-24].

Serious games are interventions where gaming elements are an integral and primary method for achieving goals related to mental health [25]. Gamification, in turn, is a technology used in digital interventions that incorporates game elements in nongame contexts (eg, reward or point systems) [26]. Both approaches have recently emerged as promising methods to enhance digital mental health technologies and increase adherence [14,18,23,27,28]. Existing serious games for adolescents typically include adventure worlds with tasks of different complexity levels and social simulations, often addressing anxiety and/or depressive symptoms. However, several important gaps remain. First, many existing interventions are disorder-specific, whereas there is a growing need for transdiagnostic approaches that address the complex and often comorbid nature of adolescent mental health difficulties [29,30]. Second, prior studies have frequently relied on relatively homogeneous samples, limiting generalizability and underscoring the need to include adolescents from more diverse school settings and socioenvironmental contexts [31-33]. Third, prior work has predominantly relied on self-report questionnaires to assess outcomes [20], indicating a need for more comprehensive evaluation approaches that incorporate additional sources of evidence, such as qualitative interview data [34], and follow-up with adult stakeholders directly connected to study participants [31]. In addition, most research on digital mental health interventions for young people has been conducted in the United States and Australia, with comparatively few studies implemented and evaluated in European settings [35]. As a result, there is limited evidence regarding the feasibility, acceptability, and effectiveness of such interventions across diverse European cultural, educational, and health care contexts. This is particularly important given that differences in school systems, service provision, and sociocultural factors may influence both engagement with and outcomes of digital interventions, necessitating contextually tailored approaches. Furthermore, there is increasing recognition—particularly within European research and policy frameworks—of the importance of involving adolescents [30,36,37] and key stakeholders (eg, parents and teachers) [38] throughout the design and implementation process to ensure relevance, usability, and sustainability. Taken together, these limitations highlight the need to develop and rigorously evaluate novel, transdiagnostic, and contextually adapted digital interventions for adolescents. The SMILE (Supporting Mental Health in Young People: Integrated Methodology for Clinical Decisions and Evidence-Based Interventions) project addresses these gaps by developing a serious game designed to support young people in building meta-skills and cognitive competencies that foster resilience to everyday stressors and enhance overall well-being.

The European project SMILE aims to promote resilience in young people by providing a gamified platform that incorporates elements of digital cognitive behavioral interventions. This platform is designed to achieve change in mental health and well-being by enhancing key psychological factors, such as cognitive flexibility, self-efficacy, critical thinking, self-regulation, and self-confidence. Within the app, algorithms will provide a gamified environment focused on mental health, including virtual self-assessments and self-monitoring, all integrated into a mobile app. The meta-skills and cognitive competencies targeted in SMILE are core components of cognitive behavioral therapy (CBT) and can be understood as transdiagnostic skills to promote adaptive coping across a range of emotional and behavioral challenges by identifying and changing maladaptive thought patterns. Although digital mental health tools have been increasingly used to improve access to mental health support, many such tools primarily focus on symptom monitoring or psychoeducation and suffer from low engagement and adherence to the interventions [39]. A contributing factor to issues of low engagement and adherence may be a lack of digital interventions available that systematically operationalize CBT principles into interactive, developmentally appropriate activities to support skill acquisition and real-world implementation. SMILE aims to address this gap by embedding the digital intervention with structured, gamified, CBT-based techniques, supported by self-monitoring and tailored feedback, to facilitate the development of these meta-skills and cognitive competencies into real-world contexts.

Additionally, SMILE will measure specific gaming behaviors, known as “digital biomarkers,” described in this protocol as in-game measures in Table 1. These measures have been previously used to assess, for example, social anxiety by collecting proximity measures and movement patterns in interaction with an avatar [40] or engagement in social scenarios [41], motor and balance skills as biomarkers for physical well-being in exergames [42], and emotional regulation skills by cheat code usage [27] or to identify nonplayer characters’ (NPC) emotions [14].

An important element of the CBT engagement and digital biomarkers within the game is understanding how reflection on these aspects enhances learning and therefore, psychological resilience. Feedback in digital mental health tools is grounded in experiential learning, cognitive behavioral models, and self-regulation frameworks, supporting users as they practice and refine coping strategies. Within serious games, game-based feedback helps users test actions, observe outcomes, and transfer these skills from digital rehearsal to real-world situations. Delayed feedback, outside of the game, can deepen reflection and understanding of thoughts and behaviors.

**Table 1.** In-game measures (“digital markers”) collected in the proof-of-concept study.

Category	Measure
Behavioral markers	Time on task Approach or avoidance count Speed of approach or avoidance
Cognitive performance	Memory: remembering instructions Decision-making: deciding which route or course of action to take under a limited time (decision-making) Speed of decision-making Attention: dwell time on NPCs^a^, comics, and other artifacts (attention) Inhibition (attention) Set shifting (cognitive flexibility) Selection of positive, negative, neutral, self-referential, or other-referential thoughts
Motor performance	Number of clicks, taps, or other interactions, and movement patterns within the game
Social biomarkers	Negative self-referential descriptions (from menu selection) Interaction with NPCs (dwell time) Proximity to NPCs Speed of approach to NPCs NPC selection Drop-off points: where users typically stop playing or lose interest
Affect	Mood and anxiety
Engagement	Brief recording of facial expression in limited exercises Duration: average length of time spent per session and per level Number of attempts at level Frequency: how often the user engages with the game Interaction with objects (frequency and duration) Content preference
Problem solving	Correct and incorrect responses—puzzles Number of attempts to correctly solve the puzzle (related to identifying maladaptive thoughts) Number of items (eg, lizards) captured

a NPC: nonplayer character.

### Study Aims

The SMILE approach places special emphasis on cocreation, defined as “the participation of users/consumers in the development process (…) significantly improving the quality, usability, and social acceptance of new solutions” [43]. Our previous studies [44] within this project included focus groups and workshops organized with adolescents and young adults in 7 participating European countries (aged 10‐24 years), as well as the Living Labs testing phase, where a first version of the game was tested by a small number of representatives from each age group (see a comparable approach with Living Labs in the study by Santonen et al [45]). The current stage of the project comprises 2 phases: a proof-of-concept study using a cluster randomized multisite multiarm adaptive trial design and key stakeholder interviews.

### Proof-of-Concept Study

The overarching aim of the proof-of-concept study is to investigate the acceptability, feasibility, and preliminary efficacy of the gamified platform designed to enhance the well-being of adolescents and young adults in a pragmatic cluster randomized multisite multiarm adaptive trial. This study has 2 primary objectives. First is to investigate feasibility and acceptability in terms of reach (ie, participation, engagement, and adherence), preliminary efficacy operationalized as greater improvement of psychological distress, well-being, and resilience posttest, in the experimental conditions compared with the control period as the primary outcome, and engagement with the gamified intervention platform. This will provide the basis for assessing the public health impact of the use and engagement with the gamified platform. To provide context for the stated hypotheses, the SMILE intervention is delivered via 2 interconnected smartphone apps: the SMILE Game App (Nurogames and DigitalTwin Technology), which provides 5 modules of interactive CBT-based gameplay, and the SMILE Companion App (RDIUP), which facilitates self-monitoring, experience sampling methods (ESMs), and the collection of study metrics. Participating clusters are randomized to 1 of 2 intervention variants: a feedback condition or a no-feedback condition. While both groups engage with identical game content and monitoring assessments, participants in the feedback condition additionally receive automated, personalized data visualizations within the SMILE Companion App. These visualizations integrate in-game metrics, daily mood, and contextual data, aiming to enhance experiential learning, self-reflection, and the transfer of acquired skills to real-world contexts. A comprehensive description of these digital tools is provided in the “Methods” section. Specifically, we expect the following:

- Hypothesis 1 (primary): compared with the control period, anxiety and depression scores (primary outcomes) will be lower in the experimental conditions (ie, with and without feedback) at posttest.
- Hypothesis 2 (secondary): compared with the control period, the secondary outcomes (well-being, resilience, emotion regulation, and self-efficacy) will be higher and social anxiety lower in the experimental conditions (ie, with and without feedback) posttest.
- Hypothesis 3 (secondary): feedback obtained in one of the experimental conditions will be positively associated with the feasibility, acceptability, and preliminary efficacy of the SMILE tools.

Our second aim is to identify contexts, psychological processes, and mechanisms of change, and how these are associated with outcomes of the intervention in participants. To this end, we will test the following hypotheses:

- Hypothesis 4 (secondary): compared with the control period and the experimental condition without feedback, well-being and resilience will be higher in the experimental condition with feedback posttest.
- Hypothesis 5 (tertiary): multimodal digital observable cues (language, speech, and facial markers), collected using diary recordings, will discriminate between children, adolescents, and young adults under mental distress and those without. Moreover, the digital observable cues will discriminate between anxiety and depression and severity.

Finally, we will conduct exploratory analysis to map digital biomarkers from in-game metrics and diaries to subjective anxiety and mood scores collated from in-game, ESM, and reflective exercises from the SMILE Companion App.

### Key Stakeholder Interviews

The aim of this part is to understand how participants leverage the SMILE intervention to support their mental health and well-being and to evaluate the process of implementing the intervention within their day-to-day contexts using a realist evaluation framework [46] to identify in vivo configurations of contexts, processes, and mechanisms of change, and how these are associated with outcomes of implementation and intervention. To achieve this, we will conduct interviews with young people who participated in the proof-of-concept study, their legal guardians, school professionals, and clinicians. The objectives are to (1) gain deeper insight into how, when, and in what contexts adolescents used SMILE tools, and how useful and engaging they perceived them to be; and (2) identify barriers and facilitators relevant to the future use and implementation of SMILE tools in educational and clinical settings.

## Methods

### Ethical Considerations

This study is conducted as a series of single-site trials that are governed locally and share a common protocol but obtain individual ethical approvals from their respective institutional review boards or ethics committees prior to commencing the study. Sites will be linked through a common protocol and measures and a cross-site data sharing agreement which will allow for combined analysis of findings. Specifically, the trial was approved by the Institute of Psychology at SWPS University in Warsaw, Poland (opinion number 23/2025); the Research Ethics Committee at the Faculty of Arts, University of Maribor (038-26-192/2024/27/FF/UM-4) in Maribor, Slovenia; University of Edinburgh School of Health in Social Science Research Ethics Committee (24-25CLPS015) in Edinburgh, United Kingdom; Heriot-Watt University Social Sciences Ethics Committee (2024-4831-13233) in Edinburgh, United Kingdom; Cyprus National Bioethics Committee (102283) in Nicosia, Cyprus; The Ethics Committee for Research with Medicaments in the Health Areas of Valladolid (PI-25‐358 O) in Valladolid, Spain; Ethics Committee of the Medical Faculty Heidelberg University (S-623/2024) in Heidelberg, Germany; and Bioethics Committee of the University of Bologna (0174183) in Bologna, Italy.

For participants who are minors, both the participant’s informed assent and the legal guardian’s informed consent are required. Only minors for whom both forms of agreement are obtained are eligible to participate in the study. The age threshold requiring legal guardian consent varies across participating countries, and the study procedures were implemented in full compliance with the respective national regulations. Specifically, in Poland, Spain, Germany, Cyprus, and Italy, parental or legal guardian consent was obtained for participants younger than the age of 18 years, whereas in Slovenia, such consent was required for participants younger than the age of 15 years, and in the United Kingdom for those younger than the age of 16 years. Participants in the proof-of-concept study do not receive financial reimbursement for their participation. In the stakeholder interview component of the study, participants in Germany will receive small book vouchers valued at €25 (approximately US $28), while no compensation is planned at other sites. Participation in the study is entirely voluntary. All participants are informed, in age-appropriate language, that they may withdraw from the study at any time without providing a reason and without any consequences.

The SMILE game aims to build resources and competencies without requiring players to disclose or explore personal negative experiences. However, gameplay may increase awareness of challenges in participants’ lives. While the game suggests coping strategies for some challenges, it does not address all possible difficulties. Participants (and legal guardians of minors) are informed in advance about available support services in their respective countries. An adverse event monitoring procedure has also been implemented. All adverse events occurring during the trial will be recorded, managed by the investigator as appropriate, and followed up. Events occurring after the completion of the study will also be documented if they are related to the intervention or study procedures. All adverse events and adverse device effects will be listed, statistically analyzed, and assessed by the sponsor for potential causal relationships with the investigation. The parties responsible for conducting the study meet regularly to monitor progress, review any adverse events, and oversee overall trial conduct. Additionally, a Data Monitoring and Ethics Committee has been established, comprising experts in Youth Mental Health, Clinical Trials, and Digital Interventions. The committee is independent from the sponsor and funder.

The proof-of-concept study was prospectively registered with the UK Clinical Study Registry (ISRCTN32523126) on April 29, 2025 by the chief investigator, before the enrollment of the first participant. A full statistical analysis plan will be registered before the end of recruitment and before data are available to the investigators. Any changes to the protocol will be transparently reported when disseminating study results.

### Data Management and Sharing

In the proof-of-concept study, personally identifiable information (PII), such as names, email addresses, and birth dates, is only stored for the purpose of adverse events handling and is never stored on any central servers. During the setup of each pilot sequence, the users are imported and a pseudonym is created. Each of the study locations individually performs this import and receives a key for mapping users to their pseudonyms. This key is stored locally by a designated investigator of the local study and is kept physically separated from any other data. This local key is only accessed for the purpose of handling adverse events. The SMILE apps, that is, SMILE Game App and SMILE Companion App (see “SMILE Game App” and “SMILE Companion App” sections below), do not store data; they only collect data according to the specifications and send them to the SMILE API for storage and processing. These data are deidentified and pseudonymized and do not contain any PII. Data in-flight are never transmitted in plain text and all connections use Transport Layer Security (TLS) for encryption. The data are stored on a server that uses the Fast Healthcare Interoperability Resources standard. Once the data are stored, access to the data is guarded by the Streaming Attribute Policy Language (SAPL) [47] engine that is part of the SMILE project, allowing for fine-grained access control. The rules for viewing the data are made based on discussions within the consortium to ensure that all parties have access only to the necessary data. Data visualization tools and analytics are available for consortium partners (based on the established rules) using the SMILE DSS (Decision Support System). All SMILE tools use the open-source solution Keycloak for federation, strong authentication, and user management.

In the stakeholder interview component, interviews will be recorded and subsequently transcribed. All transcripts will be anonymized prior to analysis, and recordings will be securely stored and permanently deleted after transcription and verification. Upon study completion, deidentified participant data, along with codebooks and all materials necessary for replication, will be made publicly available in open repositories.

### SMILE Tools: Overview

The intervention is delivered through 2 components: the SMILE Game App and the SMILE Companion App. Participants download and use both apps, which are required for participation. Both apps use the same login credentials and are linked through a single account. Both the Game and Companion apps are designed for use across age groups and in a manner that supports simultaneous accessibility for both adolescents and young adults; it was not feasible within the study constraints to provide age-specific content as originally planned.

### SMILE Game App

The game follows a player who moves to the cyberpunk city of Hopetown with their sister, Seraphina. In this world, automatic thoughts appear as small, robot-like creatures. When a cybernetic dragon attacks the city and captures Seraphina, the player embarks on a quest to rescue her. Guided by key characters, the player learns CBT-based techniques to understand and manage negative automatic thoughts. Across 5 story-driven modules, players engage in interactive challenges designed to promote cognitive restructuring, resilience, and healthier thinking patterns.

Module 1: “Hopetown.” The player investigates the city’s attack and discovers that the villain manipulates citizens’ negative automatic thoughts. Challenges introduce the CBT “hot cross bun” model [48], teaching players to distinguish between thoughts, feelings, bodily sensations, and behaviors, identify automatic thoughts, and recognize cognitive distortions.

Module 2: “Starfall District.” In a corrupted district, the player helps NPCs trapped in negative thinking cycles. This module deepens understanding of the links between thoughts, feelings, bodily sensations, and behaviors and introduces cognitive restructuring and paced breathing as tools for breaking negative cycles and strengthening emotional regulation and resilience.

Module 3: “The Technicians’ Quarters.” The player uncovers the technician’s core beliefs to unlock key information to find their sister. This module focuses on identifying and challenging core beliefs. During a fight-or-flight scenario, players must actively apply previously learned CBT-based tools to overcome negative automatic thoughts.

Module 4: “Library.” While searching for information to defeat the villain, the player evaluates evidence and tests the validity of competing narratives. This module strengthens critical thinking and evaluation skills, reinforcing cognitive restructuring and self-regulation techniques.

Module 5: “The Data Vault.” In the final confrontation, the player applies all acquired skills to defeat the villain and rescue Seraphina. A closing social scenario requires assertive communication, targeting fear of negative evaluation, reducing avoidance, and strengthening social confidence through behavioral practice.

### SMILE Variants: Feedback vs No-Feedback

Clusters of participants are randomized (see details in the “Randomization: Proof-of-Concept Study” section below) to either a feedback or a no-feedback variant of SMILE. The feedback component is expected to function as an intrinsic motivator for engagement and learning and to facilitate greater transfer of skills acquired in the game to real-world experiences. The feedback system is based on Nicholson’s [49] “Recipe for Meaningful Game Engagement” and delivered via the SMILE Companion App. Feedback is generated across time points using a triad of data sources: in-game metrics, ESMs, and self-report measures. Upon completion of each module, players receive feedback and are invited to explore specific time points of interest. The system provides brief, automated summaries (eg, “your mood appeared to be slightly more positive than earlier in the day”), linking contextual and gameplay data with daily mood and well-being ratings. The SMILE Companion App (see below for more details) presents this feedback in graphical form, integrating in-game, ESM, and reflective exercise data. Visualizations include simple daily averages, trends over time, and basic associations between activity, context, and well-being. Players can select specific indicators to explore how they relate to in-game activities, real-world experiences, and other measured variables. Participants assigned to the no-feedback condition complete the same game modules, ESM prompts, and reflective exercises, but do not have access to personalized feedback or data visualizations.

### SMILE Companion App

The SMILE Companion App supports self-monitoring, feedback, and data collection during the proof-of-concept trial. All participants are required to use the app, as baseline and first-week outcome assessments are mandatory. The app delivers self-report measures, ESM prompts, and diary entries via push notifications. Participants can individually adjust the timing of these prompts to fit their daily routines. In addition, users can customize selected features (eg, light or dark mode) and receive rewards for completing study activities. The app also provides an overview of study progress, including the current module, scheduled play periods, and pending questionnaires.

Participants assigned to the feedback condition receive personalized feedback within the app. The feedback acts as an intrinsic motivator for engagement and a link between the skills learned in the game and real-world scenarios and experiences. This feedback is delivered to only half of the sample to evaluate its effectiveness. Feedback is based on Kolb’s experiential learning model [50], Gibbs’ Reflective Cycle [51], and Nicolson’s recipe [49] for linking experiential learning from the game, feelings from the ESM measures, analysis, presented in graphical form from the in-game measures and decision-making in the cognitive restructuring exercises. Reflections on these elements are then used for real-life goal-planning strategies. The clusters of participants who do not receive the feedback will still participate in the game and ESM exercises, but will not be able to visualize and reflect on their data.

### Study Design

#### Proof-of-Concept Study

The protocol follows the SPIRIT (Standard Protocol Items: Recommendations for Interventional Trials) guidelines [52], with the checklist available in Checklist 1.

**Figure 1.**
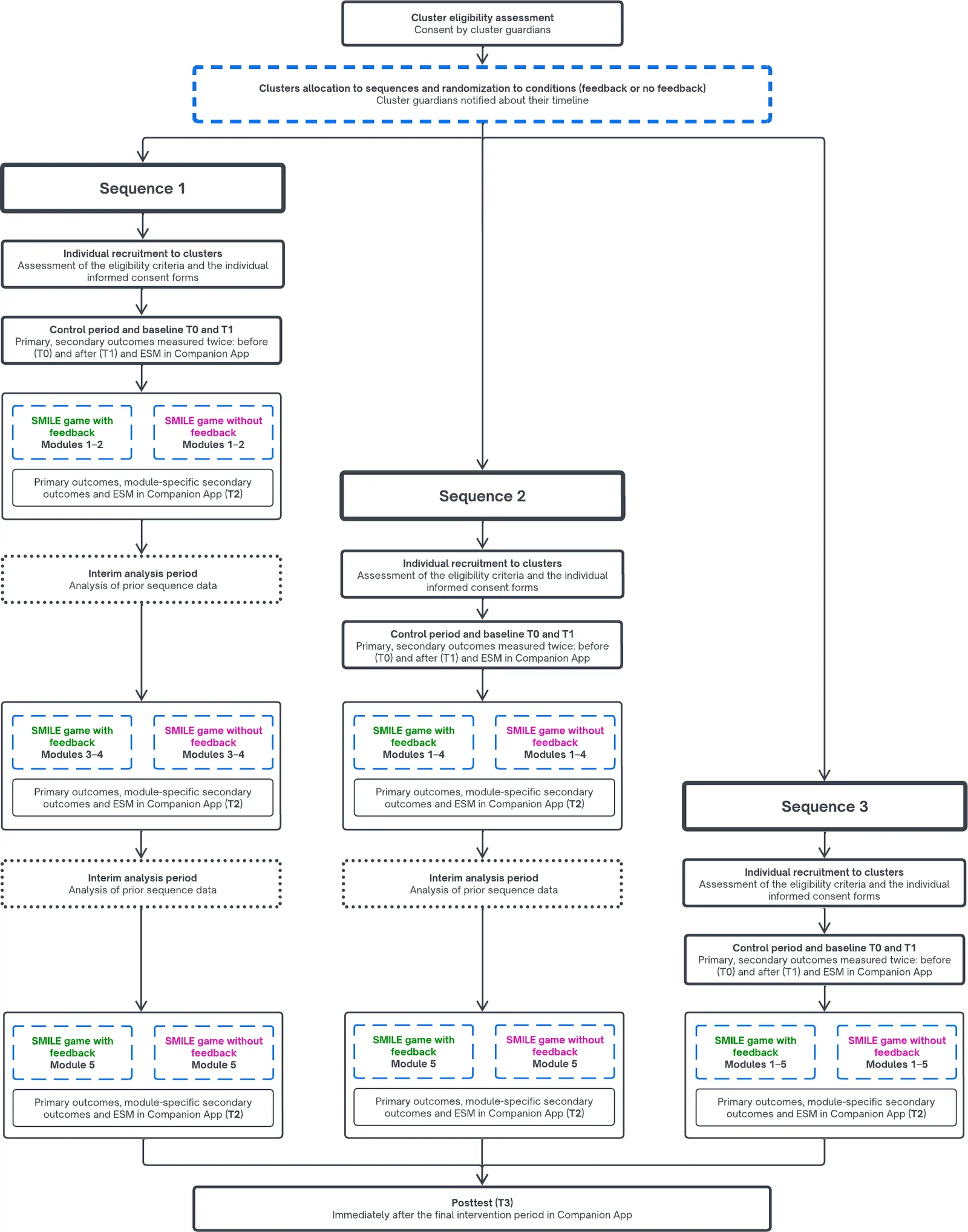
Study flowchart. ESM: experience sampling method.

This proof-of-concept study uses a cluster-randomized, multisite, multiarm adaptive trial design. Participants are grouped into clusters (see Participants Recruitment and Procedure), which are allocated to 1 of 3 sequences based on feasibility. Each cluster is subsequently randomized to either feedback or a no-feedback variant of the intervention. Clusters assigned to Sequence 1 complete a neutral baseline period with ESM, followed by Game Modules 1‐2. Clusters in Sequence 2 complete the baseline period and Game Modules 1‐4. Clusters assigned to Sequence 3 complete the baseline period and all 5 game modules (Figure 1).

The adaptive trial design is based on an initial site-level sample size calculated under the assumption that all steps in the sequence will be completed. Two stages of adaptive interim analyses will be conducted to estimate the effects of each intervention modality, including the control condition. Based on these interim results, sample sizes per cluster or site may be adjusted and decisions may be made to amend or discontinue specific intervention elements. Each adaptive interim analysis will estimate effects on the primary outcomes and identify key predictors of these effects. Sample sizes and randomization sequences may be updated accordingly. Additionally, active intervention elements may be modified based on model parameters, including patterns of feature use and the contribution of intervention-related indicators to interim effect sizes. The trial will be complemented by a process evaluation [53], using a mixed-methods realist evaluation approach. This design enables the assessment of multiple aspects of the proof-of-concept study (ie, acceptability, feasibility, reach, and efficacy). In particular, it supports the estimation of efficacy with a low risk of bias (eg, by minimizing contamination between experimental and control periods), while also allowing for in-depth process evaluation and enhancing the generalizability of findings in a naturalistic field setting.

The intervention is rolled out over a 10-month period from September 2025 to June 2026. Three sequences are planned, separated by 3-week intervals; however, the length of the interval may vary depending on any implemented modifications. Clusters are allocated to sequences based on site-specific feasibility and readiness, meaning that clusters enter the rollout at different points depending on when they are prepared to initiate the intervention. Consequently, the number of clusters varies across sequences. In total, 46 clusters across 7 pilot sites will be included in the rollout. Each game module is designed to last no longer than 20 minutes. With 5 modules in total, including all study measures, participants are expected to spend a maximum of approximately 4.5 hours on the study, distributed over several weeks.

#### Key Stakeholder Interviews

Qualitative material will be collected from semistructured in-depth interviews with adolescents, their parents or legal guardians, and school professionals after adolescents have completed the intervention period. Interviews will be conducted at each pilot site, either in person or online. Clinicians will be interviewed specifically on the topic of the SMILE DSS as part of the SMILE tools. The interview guides are presented in Multimedia Appendix 1.

### Inclusion and Exclusion Criteria

#### Proof-of-Concept Study

Participants are adolescents and young adults aged 10‐24 years who meet the following inclusion criteria: (1) provision of informed consent (participants aged <16 years in the United Kingdom, <15 years in Slovenia, and <18 years in all other pilot sites require consent from a parent or legal guardian in addition to their own assent); (2) completion of measures assessing the primary outcomes; and (3) normal or corrected-to-normal vision. Participants are excluded if they are unable to provide informed consent or if they report a current confirmed diagnosis or ongoing treatment for any of the following conditions: (1) a severe mental disorder (eg, schizophrenia, bipolar disorder, or severe depression); (2) a substance use disorder; (3) epilepsy; or (4) gaming addiction. At the University of Edinburgh site, participants were not excluded based on current mental health diagnosis or treatment status, in line with local ethical guidance. No restrictions are placed on concomitant care. The study does not monitor, regulate, or influence participants’ use of additional support, services, or interventions during the trial.

#### Key Stakeholder Interviews

For parents or legal guardians, eligibility requires having a child in their care who has completed participation in the active phase of the intervention (ie, finished playing the game). School professionals and mental health clinicians are eligible if they are currently working with adolescents and young adults aged 10‐24 years. Adolescents and young adults are eligible to participate after completing the game. No exclusion criteria apply.

### Participant Recruitment and Procedure

#### Proof-of-Concept Study

Each of the 7 participating sites implemented its own recruitment strategy while adhering to shared guidelines. Participants are recruited primarily through schools and universities, with additional outreach via private networks, social media, and community channels. Clusters are defined at the level of the recruitment setting. In most cases, a cluster corresponds to a single school or university. In addition, some “open clusters” were formed by grouping adolescents or young adults recruited from multiple sources (eg, via social media advertisements). Within each cluster, participants are enrolled up to a predetermined size, typically comprising 40‐60 individuals. Adolescents, young adults, and, where applicable, their legal guardians receive an information sheet and provide informed consent prior to participation. Eligibility is then assessed according to predefined inclusion and exclusion criteria, and eligible individuals are enrolled in the study. Before the intervention begins, participants receive onboarding materials with instructions for downloading and setting up the study apps. Depending on the site, onboarding is conducted either remotely or during a brief in-person session. At the start of the study, participants are provided with login credentials and instructions for initiating gameplay. Participation in the trial is fully remote. Participants engage with both the SMILE Game App and the SMILE Companion App at their own pace and at times convenient to them, with occasional check-ins from the research teams.

#### Key Stakeholder Interviews

During recruitment for the proof-of-concept study, participants are asked whether they would be willing to take part in a follow-up interview after completing the game; this invitation is repeated at the end of the intervention. From those who express interest, 10 adolescents per site are randomly selected and invited to participate in individual interviews. Parents or legal guardians are similarly invited following their child’s completion of the game, with 10 selected per site from those who indicate willingness to participate. Teachers from participating schools or colleges are also invited, and 5 school professionals per site are randomly selected. Where appropriate, up to 5 child and adolescent mental health professionals per site are recruited through local services, professional networks, and targeted outreach (eg, social and traditional media). Interested clinicians are contacted by the research team, provided with study information, and screened for eligibility prior to participation. Semistructured interviews are conducted after adolescents have completed the intervention. Interviews take place at each pilot site, either in person or online, and are scheduled only after participants in a given sequence have completed the game. Each interview lasts approximately 30‐60 minutes. All participants provide informed consent prior to participation.

### Sample

#### Proof-of-Concept Study

The trial will be conducted among clusters of adolescents and young adults, aged 10 to 24 years who fulfill the inclusion criteria. The sample size calculation was specified a priori assuming 3 sequences with 4 clusters per sequence. Because the sample size calculation, conducted with the Shiny CRT Calculator [54], permitted specification of only a single intervention condition, the sample size estimate was doubled to reflect the trial’s intervention structure. The calculation assumed an error level of α=.05, power of 1–β=0.80. Clustering was modeled with an intracluster correlation (ICC) of 0.01 [55,56], and within-participant autocorrelation was set to 0.60 [57]. A standardized effect size of 0.20 was assumed for all primary outcomes. Since the sample is considered to derive from a healthy population, changes in depression and anxiety symptoms were expected to be small. Applying the same effect size across outcomes provided a consistent basis for sample size estimation and represented the smallest effect of interest. Given the exploratory nature of the study, no formal alpha adjustment was applied. A dropout rate of 50% was assumed. Under these assumptions, 24 participants per arm per site are required, corresponding to 992 participants overall. On top of the included dropout expectations, to account for attrition reported in comparable trials (24%‐83%) [58,59], the recruitment target was increased to 1438 participants.

Because the trial follows an adaptive design with 2 interim analyses, the initial sample size estimate will be treated as provisional. Interim data will be used to update key parameters and adjust sample size for subsequent stages as needed. This approach will enable us to gather sufficient data to inform potential subsequent definitive randomized controlled trials and to refine the intervention as necessary.

#### Key Stakeholder Interviews

Semistructured interviews will be conducted with adolescents aged 10‐24 years who completed the SMILE intervention as part of the proof-of-concept study (n=10), as well as parents or legal guardians (n=5‐10), school professionals (n=5‐10), and clinicians (n=5‐10). In total, we intend to interview n=25 per site at a minimum and 40 at a maximum, amounting to 280 participants in total. To determine the sample size, we used the concept of information power [48,49]. We estimated sample size based on (1) the specificity of study aims, (2) the specificity of the study sample, (3) the theoretical background of the study, (4) the quality of the interviews with study participants and, finally, (5) analysis strategy (cross-case vs case), and (6) feasibility, as 4 participant groups will be coordinated in 7 pilots following one interview schedule.

### Randomization: Proof-of-Concept Study

Proof-of-concept study uses a cluster randomized multisite multiarm adaptive trial design, meaning that randomization occurs at the cluster level and involves assigning each cluster to either feedback or no-feedback variant. Randomization is conducted in blocks (using block randomization) at the consortium level using REDCap software (Vanderbilt University) by a statistician who is not involved in the study. Confirmed clusters for the upcoming sequence were randomized and this process was repeated for new clusters before each sequence. Clusters are allocated pragmatically between sites and adjusted in size and distribution if indicated by the adaptive interim analyses. There is no randomization at the individual level. Clusters will be blinded to their allocation (ie, whether they receive the intervention with or without feedback). No unblinding is planned during the trial.

### Measures in the Proof-of-Concept Study

There are 7 types of data that we will collect in this phase. First, there are self-reported primary and secondary measures that will be collected at the following time points (Table 2): baseline (T0; 1 to 2 weeks before the intervention), right before the intervention (T1), during the intervention (T2), and at posttest (T3; after completion of the intervention). Additionally, measures of depression and anxiety (ie, primary outcomes) will be distributed on a weekly basis, alternating between a depression scale 1 week and an anxiety scale the next. Secondary measures (ie, well-being and resilience) will be collected every other week. Second, participants will be invited to respond to a short daily survey using ESM, 5 times a day (Table 3). Third, there will be in-game measures related to the current activity in the game (Table 1). Fourth, we will collect vocal and facial data via weekly diary recordings. Fifth, we will assess the feasibility and acceptability of the game through measures other than participants’ responses. Finally, we will collect user data that are restricted to the SMILE apps: the Game App and the Companion App. All measurements described below will be collected by the SMILE end user apps.

**Table 2.** Study measures and time points.

Variable	T0^a^	T1^b^	T2^c^				T3^d^
	Sequence 1			Sequence 2		Sequence 3	Posttest
	Week 1	Week 2	Week 3	Week 4	Week 5	Week 6	
Anxiety	✓	✓		**✓**		**✓**	**✓**
Depression	✓	✓	**✓**		**✓**		**✓**
Well-being	✓	✓		**✓**		**✓**	**✓**
Resilience	✓	✓		**✓**		**✓**	**✓**
Privacy concerns	✓						
Acceptance (of SMILE^e^ Game App)							**✓**
Acceptance (of SMILE Companion App)							**✓**
User experience (in SMILE Game App)							**✓**
User experience (in SMILE Companion App)							**✓**

a T0=Time 0 (before baseline period).

b T1=Time 1 (before the start of the game).

c T2=Time 2 (during the game period).

d T3=Time 3 (after the intervention).

e SMILE: Supporting Mental Health in Young People: Integrated Methodology for Clinical Decisions and Evidence-Based Interventions.

**Table 3.** Data collected via experience sampling methods.

Variable	Morning	Midday	Midday	Midday	Evening
Sleep quality	✓				
Mood		✓	✓	✓	✓
Location		✓	✓	✓	✓
Activity context		✓	✓	✓	✓
Social context		✓	✓	✓	✓
Stress coping		✓	✓	✓	
Emotion regulation		✓	✓	✓	✓
Reflective functioning					✓
Social anxiety^a^		✓	✓	✓	
Self-efficacy^a^					✓

a Collected only during baseline and then again in weeks 5 and 6.

### Primary Outcomes

Primary outcomes are depression and anxiety. To measure anxiety, we use the Penn-State Worry Questionnaire for Children (PSWQ-C [60]) for participants aged 10‐17 years and the Generalized Anxiety Disorder-7 item (GAD-7 [61]) for participants aged 18‐24 years. To assess depression, we use the Patient Health Questionnaire for Adolescents (PHQ-A; [62]) for participants aged 10‐17 years and the Patient Health Questionnaire-9 item (PHQ-9; [63]) for participants aged 18‐24 years. Completion of primary outcome measures at T0 and T1 is mandatory within the SMILE apps.

### Secondary Outcomes

Main secondary outcomes include self-reported well-being and resilience, assessed with measures appropriate to the age group: the Warwick–Edinburgh Mental Well-being Scale (WEMWBS [64]) for ages 10‐24 years, the Child and Youth Resilience Measure (CYRM-12 [65]) for ages 10‐17 years, and the Brief Resilience Scale (BRS [66]) for ages 18‐24 years. Moreover, we measure online privacy concerns [67], technology acceptance [56], and user experience for both apps using the short User Experience Questionnaire [68]. Completion of secondary outcome questionnaires is optional for participants.

In addition to questionnaire data, selected secondary outcomes will also be derived from daily ESM responses (Table 3).

### Other Measures

In addition to the primary and secondary outcomes, we will assess feasibility and acceptability outcomes, user data, in-game metrics, and diary recordings.

Feasibility and acceptability outcomes will include (1) cluster recruitment yield, defined as the proportion of clusters included relative to the total number of clusters invited, with a predefined success criterion of at least 80%; (2) uptake, defined as the proportion of participants who completed the minimum intervention dose (defined as completing baseline measurement and at least one game module) relative to all included participants, with a predefined success criterion of at least 66%; (3) engagement with the SMILE serious game based on in-game measures (Table 1); (4) adherence, operationalized as the average proportion of completed game modules relative to the total of 5 modules, with a predefined success criterion of at least 50%; (5) dropout, defined as the proportion of participants who disengaged from the study at any point between baseline and posttest relative to all participants, with a predefined criterion of less than 45%; (6) adverse events, assessed and summarized.

We will also collect user data restricted to the study apps (ie, the Game App and Companion App). User data will include app usage frequency; drop-off points (where users typically stop using the app); session duration; session start and end times; whether sessions are completed with or without interruptions; completion rates; frequency of engagement with the game; and the average time spent per session.

During SMILE gameplay, the Game App will collect various in-game metrics. These include measures of behaviors such as proximity to NPCs, performance in specific tasks, or in-game affect ratings. Table 1 provides a detailed description of these metrics.

As part of the intervention, in the SMILE Companion App, participants will be invited to complete brief weekly diary video recordings (approximately 3‐4 prompts) and longer recordings at baseline and posttest (approximately 8 prompts). Baseline and posttest prompts are general (eg, self-perception, mood and energy, coping, self-esteem, and potential anxiety or depression symptoms), while weekly prompts are tied to the current game module and ask participants to reflect on their experience. Recordings are encrypted and uploaded; audio and video streams are separated on the server and are not viewed or listened to by humans. Data remain encrypted until automated processing, and speech transcriptions are not stored. AI-based processing will extract exploratory digital markers previously linked in the literature [68-70] to anxiety and depression, including facial movement features (eg, action units, gaze, and head movement metrics) and acoustic or text-derived features (eg, energy and other signal parameters; counts of words and sentences and symptom-related terms), to examine associations with validated self-report outcomes and inform development of an explainable screening approach in young people.

### Measures in Key Stakeholder Interviews

Semistructured interviews with adolescents who have completed the SMILE intervention, as well as with their parents or legal guardians, school professionals, and clinicians, will cover (1) the main concerns of children and young people; (2) perceived resources and coping supports; (3) feedback on the game from parents and school professionals; (4) perceived barriers to implementing SMILE tools at home and in school; and (5) perceived facilitators to implementing SMILE tools at home and in school.

### Process Evaluation

We will conduct a process evaluation to examine the implementation of the SMILE intervention and to understand what works, for whom, under what circumstances, and why. Quantitative data will describe outcome patterns and assess implementation and intervention fidelity, while qualitative data will help explain the underlying processes shaping these patterns. Using a mixed-methods realist evaluation approach[23], we will examine how contextual factors, implementation mechanisms, and outcomes interact within the adolescent population, as well as how broader socioeconomic and environmental factors influence engagement and decision-making. This approach will also enable us to explore how adolescents adapt SMILE to meet their individual needs.

### Data Analysis Plan

#### Proof-of-Concept Study

Hypotheses regarding the preliminary efficacy of the SMILE interventions will be examined using linear mixed-effects models (LMMs), fitted separately for each primary and secondary outcome. For outcomes assessed using age-specific scales, raw scores will be transformed to z scores or percentages to facilitate comparability across age groups. Models evaluating linear time-on-treatment effects will include fixed effects of time (calendar time and exposure time), condition (game with feedback, game without feedback, and baseline control), and the exposure time by condition interaction. We will also consider models including age and gender as covariates, with the final specification determined on the basis of model comparison. The random-effects structure will reflect the hierarchical design, with 3 levels: country, cluster, and individual. To account for outcome variability over calendar time, we will additionally include random effects for the cluster by calendar time and individual by calendar time components. Analyses will be conducted on the minimum-dose intention-to-treat sample, defined as participants who complete baseline assessments and at least one game module. Given the exploratory nature of this pilot, *P* values will be interpreted descriptively.

Adaptive interim analyses will be performed at the end of each sequence to estimate the empirical effects on primary and secondary outcomes. Predefined criteria for adjustment include potential changes to the primary outcomes, sample size, and intervention elements. First, if limited variance is observed in the primary outcomes of depression and anxiety, for example, if more than 80% to 90% of participants score within the top or bottom 10% of the scale, we will consider promoting well-being outcomes to the primary outcomes. This change would be accompanied by a recalculation of the required sample size based on the expected effect size for the revised primary outcome. Second, interim results may be used to adjust the planned sample size, including the number and/or size of clusters. Third, we will consider adaptations to intervention elements based on interim findings on engagement and the delivery of feedback, including changes to game modules or modes of interaction and adjustments to the level or detail of feedback provided in subsequent intervention sequences.

#### Key Stakeholder Interviews

Interviews will be audio-recorded and transcribed. Qualitative data will be analyzed using qualitative data analysis software (eg, MAXQDA [VERBI Software] 2022 [71]). We will apply an inductive thematic analysis [72], involving familiarization with the data, systematic coding of relevant segments, identification of candidate themes, and iterative review and refinement of themes against the coded extracts and the full dataset. Themes will then be defined and named to produce a coherent thematic account. Analyses will first be conducted within each pilot site to generate site-level themes reflecting participants’ needs, experiences, and perceived best practices. These findings will subsequently inform a cross-national synthesis. An English-language report will be produced to summarize the results and support the interpretation of study outcomes and future implementation planning. Finally, quantitative data will include demographic characteristics, which will be summarized using descriptive statistics.

## Results

As of March 2026, a total of 46 clusters have been recruited, and 2 sequences have been completed. The third sequence commenced in the final days of March 2026, with clusters planned to join on a rolling basis. Primary results are expected in September 2026.

## Discussion

The SMILE project contributes to ongoing global efforts to improve the mental health and well-being of adolescents and young adults through scalable digital interventions. By delivering the intervention through a gamified smartphone app, the project aims to increase accessibility and reduce barriers to participation [6,10,14,18,23,28]. Smartphones are widely used by adolescents and young adults, and gamified environments are familiar to this population. Leveraging these existing habits may facilitate engagement and support implementation in real-world settings. The intervention is grounded in CBT principles and focuses on strengthening personal resources and coping strategies. This approach allows the program to be implemented as a universal intervention across diverse demographic and sociocultural contexts. Gamification may also help reduce some of the demands associated with traditional digital mental health interventions [73]. Presenting intervention content through intuitive game mechanics may facilitate participation among users with varying levels of cognitive and digital literacy. Furthermore, by conducting the study across 7 European countries, the SMILE project may provide insights relevant for broader implementation in Europe. SMILE has been cocreated through a multistep process that included initial focus groups with adolescents and young adults, their parents, as well as school and clinical professionals. This was followed by a Living Labs testing phase [74], during which target users provided feedback on the intervention prototype. Such a participatory approach was intended to support the development of an intervention that is relevant, usable, and engaging for the target population. The stage of SMILE described in this protocol represents a continuation of this iterative development process, focusing on feasibility and acceptability, alongside preliminary efficacy, to inform further refinement of the intervention. The results of the study will be disseminated to multiple stakeholder groups. Findings will be reported in trial registries and shared through peer-reviewed publications and conference presentations targeting the scientific and health care communities. Where appropriate, summaries of the results will also be made available to participants and the broader public in accessible, plain-language formats.

Several limitations should be acknowledged. First, recruitment followed a pragmatic approach, and participating schools were primarily selected based on availability. Consequently, the study samples may not be fully representative of the broader populations within the participating countries. Second, in addition, due to time constraints, not all feedback collected from participants during adaptive interim analyses will be implemented within the study phase. Third, SMILE is implemented as an unguided intervention and primarily relies on automated reminders from the SMILE Companion App to prompt participants to complete study measurements and progress through the game. Additional reminders encouraging participants to log into the app are sent via email by the research teams at each study site. While this approach supports the accessibility and scalability of the intervention, it may also increase the risk of participant dropout. Fourth, the study relies on the assumption that all participants have access to mobile devices for the duration of testing. While some pilot sites (United Kingdom and Slovenia) were able to offer devices to participants to facilitate participation, the study will likely not be fully representative of the broad range of digital literacy in adolescents and young adults as a result. Finally, although some objective measures are included, the primary outcomes rely on self-reported data, which may be subject to reporting bias.

In conclusion, if SMILE is found to be feasible, acceptable, and to show preliminary efficacy, it may offer a scalable and accessible tool to support the mental health and well-being of adolescents and young adults. Such an intervention could be implemented either as a stand-alone resource or as part of broader support systems, including school-based or health care–based mental health services.

## Supplementary material

10.2196/96292 Multimedia Appendix 1 Interview guides.

10.2196/96292 Checklist 1 SPIRIT checklist.

## Abbreviations

BRS: Brief Resilience Scale
CBT: cognitive behavioral therapy
CYRM-12: Child and Youth Resilience Measure
DALY: disability-adjusted life year
DSS: Decision Support System
ESM: experience sampling method
GAD-7: Generalized Anxiety Disorder-7 item
ICC: intracluster correlation
LMM: linear mixed-effects model
NPC: nonplayer character
PHQ-9: Patient Health Questionnaire-9 item
PHQ-A: Patient Health Questionnaire for Adolescents
PII: personally identifiable information
PSWQ-C: Penn-State Worry Questionnaire for Children
SAPL: Streaming Attribute Policy Language
SMILE: Supporting Mental Health in Young People: Integrated Methodology for Clinical Decisions and Evidence-Based Interventions
SPIRIT: Standard Protocol Items: Recommendations for Interventional Trials
TLS: Transport Layer Security
WEMWBS: Warwick–Edinburgh Mental Well-being Scale
WHO: World Health Organization
