# Supplementary material for: The Preliminary Efficacy and Feasibility of SMILE Serious Game for Adolescents and Young Adults: Protocol for Cluster Randomized Multisite Adaptive Trial Design
Source: JMIR Res Protoc. 2026 Sep 3;15:e96292. doi: 10.2196/96292 (PMC13541232; doi:10.2196/96292)
Supplement: Supplementary file 1 [file resprot-v15-e96292-s001.pdf]

Multimedia Appendix 1

This appendix includes four interview guides:

1. Interview guide for adolescents and young adults who used the SMILE tools.
2. Interview guide for parents or legal guardians of adolescents who used the SMILE tools.
3. Interview guide for school professionals working with adolescents who used the SMILE tools.
4. Interview guide for clinicians who routinely work with adolescents and young adults.

INTERVIEW GUIDE – Adolescents and young adults

General recommendations for using this guide:

- the following guide is intended to structure the qualitative interview
- the order of the questions can be adapted to the flow of the conversation
- it is not a matter of repeating the questions exactly; what is important is that the other person understands the question and easily starts talking
- other questions that are not listed here may also arise from the course of the conversation
- not all questions need to be asked and sometimes the probes are not needed either; participants may have already discussed a topic without being asked/additional prompting

The guide is made up of main questions (grey cells) and possible follow-ups (white cells). The main questions are intended to encourage the other person to report openly and freely. The probes/follow-ups are optional and can be asked in addition to generate relevant information that has not yet been mentioned.

1	Have you used any mental health or wellbeing apps before? Which ones?
2	Have you ever used any game-based interventions? Which?
3	How did you find using the SMILE apps?
	• What did you enjoy about it? Were there functions or content that were helpful? If so, which ones?<br>• What would you improve about the apps? Were there functions or content that were annoying or inappropriate? If so, which ones?
4	How often and for how long did you play the SMILE game? And what about the Companion App?
	• Where do you play it most? At home, school, bus?
5	How did using the apps fit in with your daily life?
	• Was it easy? Was it difficult? Why?<br>• What helped you keep going? What made you drop off? (e.g., reminders, teacher/parent/clinician support, peers)<br>• Did you have support from anyone (teacher, friend, parent) when using it?
6	Did you come across any bugs or confusing parts? How did you handle them?
7	What did you feel or think when playing the SMILE game?
	• How does it make you feel/feel differently after?
8	What did you find were the main things in your experience that the SMILE game was focusing on – name 2!
	• There were a lot of tasks in the game focused on recognising and changing thoughts. What went through your mind when completing them?
9	Which game features did you find helpful – if any? And what did they help you with?
	• Did any game features have an impact on your mood or stress?

10	**[only if participant was in the feedback condition]**<br><br>In the Companion App, you had access to feedback on your game play and questionnaire answers – how did you find it?
	• Was it helpful in any way? Or no? Why?<br>• When reading it, how did it make you feel?<br>• How did it affect you?
11	What, if anything, has changed for you since using the game?
	(e.g., mood, coping with stress, sleep, school/work, friendships, confidence, help seeking, sense of wellbeing)<br><br>• Why do you think that's the case? What made the difference?
12	Has anything got worse or been unhelpful?
	(e.g., more screen time, data use, worry, conflict)<br><br>• Tell me about a time it really helped. And a time it didn't.
13	If you could change something about the SMILE apps to make them work better for young people's mental health, what would you change and why?
14	What else do you think we need to know, to really understand how the SMILE apps have worked for you?
15	Is there anything we haven't talked about that feels important? Is there anything you want to add?

Interview guide for parents of children and young people participating in the SMILE pilot

General recommendations for using this guide

- the following guide is intended to structure the qualitative interview
- both the order of the questions and their weighting can be variably adapted to the flow of the conversation during the course of the interview
- it is not a matter of repeating the questions exactly; what is important is that the other person understands the question and easily starts talking
- other questions that are not listed here may also arise from the course of the conversation

The guide is made up of main questions (grey cells) and possible follow-ups (white cells). The main questions are intended to encourage the other person to report openly and freely. The follow-ups are asked in addition to generate relevant information that has not yet been mentioned.

General introduction:
Thanks for participating, for the willingness of your child/young person to take part in the study. Explanation of the main aims of the study.

1	What is your general opinion about using digital media for mental health?
	As an example, there is a growing number of smartphone applications available for the sake of mood tracking or mental health exercises, aiming at relaxation, coping, or resilience building.<br>• What are your experiences with using such solutions for mental health?<br>• Tell me, what encourages you to use such solutions for you and your family? Do you see any (potential) benefits? If so, what are they?<br>• Tell me, what worries you in this context? Do you see any risks or obstacles for you and your family? If so, what are they?<br>• Based on your and your family's experience, do you feel like age impacts how someone interacts with such solutions? If yes, in what way? If no, why?<br>• Finally, do you feel like gender impacts how someone interacts with such solutions? If yes, in what way? If no, why?
2	What do you think, in which areas do your children need most support?
	• Based on your experiences as a parent, what do you see as the main challenges that children and adolescents face these days?<br>◦ Do you see that in your child/children as well? What does that look like?<br>◦ How do you see your children coping with those challenges?<br>◦ What kinds of support is available to them to cope with those challenges?<br>◦ What, in your experience prevents them from seeking support? Are there any key obstacles?
3	How do you generally deal with digital media in your family?
	• What role do digital technologies (such as mobile devices, wearables, notebooks, play stations) play in your family's day to day life? How does your family approaches different digital technologies available out there?<br>• What are the gaming habits in your family? Do you game by yourself?

	• Are there any digital restrictions in your family, for example regarding ownership of devices, access to Wifi etc.? Are there time limits you set for yourself or the kids?<br><br>Give an introduction to SMILE aims… leading to question 3
4	Your son / daughter was an active participant in the SMILE study. Did you talk with him / her about the SMILE apps?
	• Tell me about their experience.<br>• What functions or content did your son/daughter find particularly helpful?<br>• Do you agree? If not, why?<br>• Were there functions or content that your son/daughter disliked?<br>• Do you agree? If not, why?
5	Did you observe any changes in your child since they started using the SMILE apps?
	• Tell me about these changes, you observed.<br>• How do you see those changes? Where they for the better, for the worse?<br>o (Did sth. similar occur before in another context?)
6	How did the use of the SMILE apps fit into your family's daily routines?
	• Please tell me about your family's daily routines and how they were affected by the SMILE apps. What was helpful to integrate them into your family's daily routines? What helped your child engage regularly with the SMILE apps?<br>• Please tell me about the obstacles you encountered? What made it more difficult for your child to engage with the SMILE apps?<br>• Tell me about your vision of a future version of the SMILE apps? What would be different in a way that they would fit into your family's daily routines?
7	Have you had a chance to take a look at the game yourself?
	• Tell me about your personal experiences and impression?<br>• What elements did you see as useful or enjoyable?<br>• What can we improve in a future version?
	Interview Wrap-up:<br>Is there anything else we didn't discuss that you think should be mentioned? Is there anything else you'd like to add?

Thank you very much for your participation!

Interview guide for school professionals working with children and young people participating in the SMILE pilot

General recommendations for using this guide

- the following guide is intended to structure the qualitative interview
- both the order of the questions and their weighting can be variably adapted to the flow of the conversation during the course of the interview
- it is not a matter of repeating the questions exactly; what is important is that the other person understands the question and easily starts talking
- other questions that are not listed here may also arise from the course of the conversation

The guide is made up of main questions (grey cells) and possible follow-ups (white cells). The main questions are intended to encourage the other person to report openly and freely. The follow-ups are asked in addition to generate relevant information that has not yet been mentioned.

General introduction:

Thanks for participating, for the willingness of your school to take part in the study.
Explanation of the main aims of the study.

1	What is your general opinion about using digital media for mental health?
	As an example, there is a growing number of smartphone applications available for the sake of mood tracking or mental health exercises, aiming at relaxation, coping, or resilience building.<br>• What are your experiences with using such solutions for mental health?<br>• Tell me, what encourages you to use such solutions for adolescents? Do you see any (potential) benefits? If so, what are they?<br>• Tell me, what worries you in this context? Do you see any risks or obstacles for adolescents? If so, what are they?<br>• Based on your experience, do you feel like age impacts how someone interacts with such solutions? If yes, in what way? If no, why?<br>• Finally, do you feel like gender impacts how someone interacts with such solutions? If yes, in what way? If no, why?
2	What do you think, in which areas do your pupils need most support?
	• Based on your experiences as a teacher, what do you see as the main challenges that children and adolescents face these days?<br>◦ Do you see that in some of your pupils as well? What does that look like?<br>◦ How do you see your pupils coping with those challenges?<br>◦ What kinds of support is available to them to cope with those challenges?<br>◦ What, in your experience prevents them from seeking support? Are there any key obstacles?
3	How does your school generally deal with mobile devices during school time?
	• Tell me how you envision fitting those to school routines / tell me about your school's policy around phones.

	• Is gaming a topic in your class / school?<br>• What role could a school take in the process of their pupils engaging in digital mental health games?
4	Some of your pupils were active participants in the SMILE study. Did you talk to them about the SMILE study?
	• What did they tell you about it?<br>• What functions or content did they find particularly helpful?<br>• Do you agree? If not, why?<br>• Were there functions or content that your students found disturbing or inappropriate?<br>• Do you agree? If not, why?
5	Did you observe any changes in your pupils?
	• Tell me about these changes, you observed.<br>• Do you value them as positive or rather critical?<br>○ (Did sth. similar occur before in another context?)
6	Have you had a chance to take a look at the game yourself?
	• If so, what experiences did you make, what was your impression?<br>• What elements did you see as useful or enjoyable?<br>• What can we improve in a future version?
	Interview Wrap-up:<br>Is there anything else we didn't discuss that you think should be mentioned? Is there anything else you'd like to add?

Thank you very much for your participation!

Interview guide for clinicians participating in the SMILE pilot

General recommendations for using this guide

- the following guide is intended to structure the qualitative interview
- both the order of the questions and their weighting can be variably adapted to the flow of the conversation during the course of the interview
- it is not a matter of repeating the questions exactly; what is important is that the other person understands the question and easily starts talking
- other questions that are not listed here may also arise from the course of the conversation

The guide is made up of main questions (grey cells) and possible follow-ups (white cells). The main questions are intended to encourage the other person to report openly and freely. The follow-ups are asked in addition to generate relevant information that has not yet been mentioned.

General introduction:

Thanks for participating, for the willingness to take part in the study.
Explanation of the main aims of the study.

1	Can you tell us some details about your professional background?
	• Describe the group of patients you are in charge for / working with<br>• Are you working in an inpatient or in an outpatient setting?<br>• What psychotherapeutic background do you have? (psychodynamic, cognitive behavioural, else…)
2	In your daily work, do you use digital mental health applications?
	• If yes, please describe the kind of application (browser based programme, smartphone app, virtual reality etc.)<br>• Please tell us about your experience when using these formats.<br>o Did you find it successful, where there any problems in using these formats?<br>o How is/was the uptake and acceptance of the patients?<br>• If not, what do you think in general about providing mental health support in a digital format?<br>• Please tell us about your experiences with applications using AI in general. Are there any tools you use regularly and why do you use them?<br>• Shifting to AI in clinical practice specifically, please describe your general attitude toward the use of AI in clinical practice.
3	In SMILE, we developed a decision support system. Decision Support Systems are digital tools that analyze data and give recommendations to assist in decision-making . Very simple examples are recommendations for online shopping based on previous behaviour or applications recommending hiking tracks based on weather. In clinical practice, it could be a system that sends an alert when a patient's values fall outside a safe range, or a tool that highlights unusual patterns in a patient's data that might otherwise go unnoticed.<br><br>Did you ever use something. similar? If yes, please describe.
	• Have you ever used any tool that processed patient data to provide you with alerts, recommendations, risk scores, or guidance to support your clinical decisions? Please tell us about your experience.<br>• Was it related to mental health?

	• In SMILE, we collect different types of data from young participants through mobile applications. Our Decision Support System has two main functions: helping clinicians explore this data, and, at a deeper level, explaining the data patterns behind mood changes in young users. Have you ever used any tool to explore or visualize patient data?
	Video input: The SMILE DSS
4	Which benefits you see in a clinical DSS, and specifically in the SMILE DSS you saw in the video, for your daily work? If any, please describe them.
	• At what point in care would you most benefit from a DSS?<br>• Are there specific benefits for certain groups of your patients?<br>• In what kind of decisions would you expect support from the DSS?
5	Please describe what you would need in order to work properly with a DSS like the one described in the video.
	• How does a DSS ideally integrate with your workflows?<br>• Would the technical infrastructure available in your ward (e.g., computers, internet access) allow you to use a DSS like the one described?<br>• Do you think that with the appropriate training you would use a system like this in your daily practice?
6	Please describe your overall impression of the design and presentation of the DSS.
	• Did the navigation and structure seem intuitive?<br>• How clear did the wording, visual design, or level of detail seem to you from the video?<br>• What information felt missing or underexplained?<br>• Did anything in the DSS you saw feel overwhelming or confusing?
7	Please describe how confident or trusting you feel toward the system outputs after seeing the video.
	• Did watching the video give you confidence in the system, and if so (or if not), what influenced this? Please describe what influenced your perception.<br>• Do you feel you understand how the system produces its outputs or recommendations?<br>• Did the functionalities shown help you judge when the system should or should not be relied on?<br>• Would you feel comfortable relying on this system in real clinical scenarios?
8	Please describe what would convince or prevent you from using this DSS in your daily work.
	• Which features would be most valuable in your day-to-day practice?<br>• What features would you like to see in this DSS to perfectly match your daily work?<br>• Are there any decisions that you would never leave to a DSS?<br>• What concerns or limitations stand out to you?
9	Interview Wrap-up:<br>Is there anything else we didn't discuss that you think should be mentioned? Is there anything else you would like to add?

Thank you very much for your participation!
